# Fluorescent Proteins for Investigating Biological Events in Acidic Environments

**DOI:** 10.3390/ijms19061548

**Published:** 2018-05-23

**Authors:** Hajime Shinoda, Michael Shannon, Takeharu Nagai

**Affiliations:** 1 Department of Biotechnology, Graduate School of Engineering, Osaka University, 2-1 Yamadaoka, Suita 565-0871, Japan; 4nd40@sanken.osaka-u.ac.jp; 2 The Institute of Scientific and Industrial Research, Osaka University, 8-1 Mihogaoka, Ibaraki 567-0047, Japan; mi9@sanken.osaka-u.ac.jp

**Keywords:** fluorescent protein, GFP, acidic organelle, lysosome, secretory granule, endosome, pH, pH-sensitivity, Ca^2+^, FRET

## Abstract

The interior lumen of acidic organelles (e.g., endosomes, secretory granules, lysosomes and plant vacuoles) is an important platform for modification, transport and degradation of biomolecules as well as signal transduction, which remains challenging to investigate using conventional fluorescent proteins (FPs). Due to the highly acidic luminal environment (pH ~ 4.5–6.0), most FPs and related sensors are apt to lose their fluorescence. To address the need to image in acidic environments, several research groups have developed acid-tolerant FPs in a wide color range. Furthermore, the engineering of pH insensitive sensors, and their concomitant use with pH sensitive sensors for the purpose of pH-calibration has enabled characterization of the role of luminal ions. In this short review, we summarize the recent development of acid-tolerant FPs and related functional sensors and discuss the future prospects for this field.

## 1. Introduction

For cells to maintain their function, the construction and transportation of useful biomolecules must be balanced with the degradation and recycling of unnecessary ones. Acidic organelles (e.g., endosomes, secretory granules, lysosomes, plant vacuoles, etc.) play an important role in biomolecule degradation. Such organelles maintain luminal pH (pH ~ 4.5–6.0), and contain many unique modification and degradation enzymes that actively work in acidic conditions [1]. In addition to these well characterized functions, their involvement in the cell-wide signaling network has recently been elucidated [2,3]. In particular, Ca^2+^ release from and uptake into acidic organelles (in concert with other Ca^2+^-storing organelles, mainly the endoplasmic reticulum) has been shown to control local Ca^2+^ levels in the cytosol, leading to the regulation of biological events such as autophagy, apoptosis and membrane repair [4]. Intraluminal Cl^−^ acts as a major counterion during the V-ATPase dependent acidification of endocytic and secretory organelles. It has also been reported that the disturbance of Cl^−^ transport into/out of vesicles leads to impaired membrane-trafficking [5]. Additionally, levels of amino acids in lysosomes control activity of the mTOR complex 1 (mTORC1) on the lysosomal membrane, regulating the autophagic pathway to control biosynthesis and the catabolic state [6].

Fluorescent proteins (FPs) are some of the most useful tools to investigate these cellular functions [7]. They enable us to observe the spatial dynamics of biomolecules and have been adapted to sense ions (for example Ca^2+^, Mg^2+^, Zn^2+^, K^+^, Cl^−^) in living cells, all of which are highly important for signal transduction. FPs and FP-based sensors can easily be localized to acidic organelles by fusing them to signal peptides or organelle-specific proteins. However, the application of FPs to study the lumen of acidic organelles has been limited because: (1) many FPs that are practical for neutral pH imaging tend to lose fluorescence in acidic conditions due to their neutral p*K*_a_ of ~6.0 [8]; and (2) functional domains used in most FP-based sensors change their affinity to target molecules depending on pH, complicating data analysis in acidic organelles in which pH is variable and sometimes dynamic. To overcome these limitations, acid-tolerant FPs have been developed, and FP-based sensors have been used in combination with pH sensors to compensate for effects triggered by pH change. In this short review we introduce the recent development of acid-tolerant FPs and compare mechanisms of pH sensitivity in conventional FPs with acid-tolerant ones. We show examples of the application of FP-based sensors in acidic organelles and discuss the future prospects for this field.

## 2. GFP-Like Protein

### 2.1. Green-Yellow-Red Fluorescent Protein

**Figure 1:**
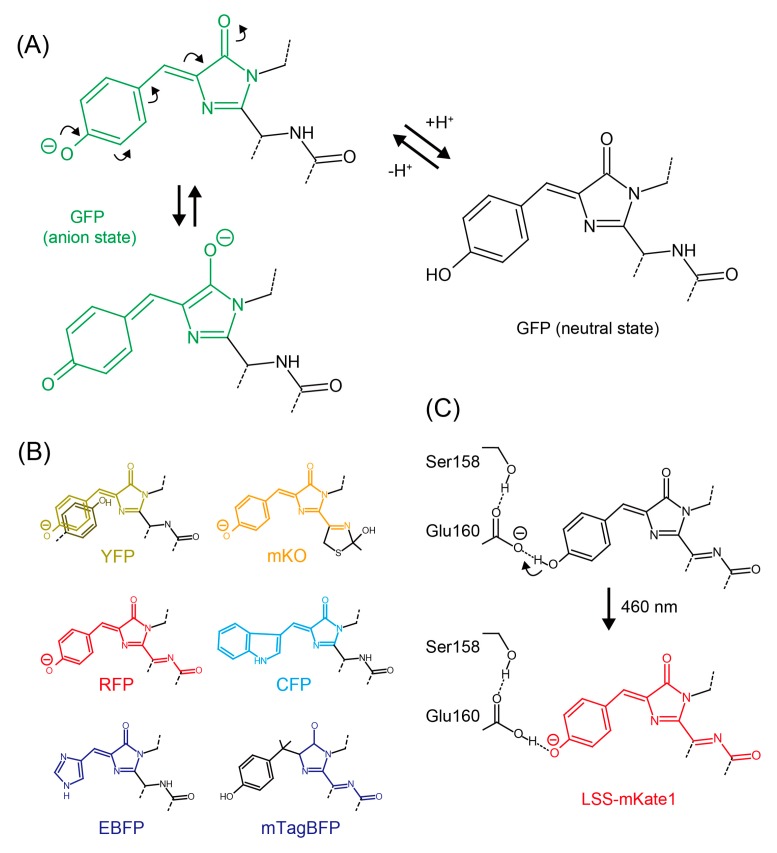
Chromophore structures of GFP-like proteins. (**A**) Protonation of the GFP chromophore causes contraction of the π conjugation system, resulting in an absorption spectral shift from 480–500 nm to 360–380 nm. The protonation also makes the chromophore unstable and results in a fluorescence quantum yield decrease; (**B**) Chemical structures of selected GFP-like proteins. The π-conjugation system responsible for fluorescence emission are colored to correspond with the color of fluorescence emission; (**C**) Excited state proton transfer (ESPT) in LSS-mKate1. Glu160 act as a proton acceptor in ESPT.

Green and yellow FPs are widely used by researchers because of their brightness, fast and simple chromophore maturation mechanism, and excellent photostability. GFPs form a *p*-hydroxybenzylideneimidazolinone chromophore, containing a hydroxyphenyl ring originating from a side chain of Tyr. YFP is a slightly red-shifted variant of GFP, a shift that is caused by a π-π stacking interaction between the chromophore and a neighboring hydroxyphenyl ring from a Tyr side chain [7]. At neutral pH, a phenolate oxide of the GFP or YFP chromophore is deprotonated by hydrogen bonding with a neighboring amino acid (usually an OH group of a side chain of Ser or Thr) and a water molecule. In this situation an electron can be reversibly relayed between the phenolate oxide ion and imidazole ring to form an imidazole oxide ion. This results in the extension of a π conjugation system and resultant light energy absorption with a peak at around 480–500 nm. Additionally, the hydrogen bonds at the phenolate oxide stabilize the chromophore by limiting the movement caused by thermal fluctuation, enabling effective energy conversion of the chromophore as it relaxes from the excited state and emits fluorescence. On the other hand, in acidic pH the phenolate oxide is protonated, which causes contraction of the π conjugation system and a shift in absorption to the UV spectral region (a peak at around 360–380 nm) (Figure 1A). Absence of a hydrogen bond at the phenolate oxide increases thermal fluctuation of the chromophore, so that excited state energy in the chromophore is likely to be consumed by thermal relaxation, leading to a decrease in the fluorescence quantum yield. Because of this acid-quenching mechanism, most widely-used GFPs and YFPs are pH sensitive (p*K*_a_ for EGFP: 6.0, mEmerald: 6.0, sfGFP: 5.5, mNeonGreen: 5.7, mClover: 5.9, mCitrine: 5.7, mVenus: 6.0 [8], Table 1).

Several wild-type GFPs less sensitive to pH such as CpYGFP (p*K*_a_ = 4.4) [9], bfloGFPa1 (p*K*_a_ = 4.0) [10] and zFP506 (p*K*_a_ < 4.0) [11] were originally reported, however their application to imaging has been limited. This is because they form homo-dimeric or -tetrameric structures under physiological conditions and/or form aggregates in cells, which can cause dysfunction or mislocalization of the fused proteins. Recently, a monomeric and bright acid-tolerant GFP: Gamillus, has been developed from flower hat jellyfish (*Olindias formosa*) [12]. Gamillus emits fluorescence with the same brightness and photo-physics over a wide pH range of 4.5 to 9.0, covering almost all physiological pH values observed inside cells (p*K*_a_ = 3.4). Mechanistically, it has been suggested that the unique *trans* configuration of the chromophore phenyl ring is important for acid-tolerance. Gamillus exhibits a photochromic decrease in fluorescence to 60% or 10% of its initial intensity when excited by 457–487 nm or 488–512 nm, which is rapidly recovered by subsequent excitation with 352–388 nm light. On the other hand, it shows a negligible photochromic decrease when excited by 440–480 nm light. Therefore, with care and caution to avoid undesired photochromic effects, Gamillus will help researchers to perform multicolor imaging or FRET analysis to investigate biological events inside acidic cellular environments.

Red FPs derived from GFP-like proteins form chromophores containing the same hydroxyphenyl ring as in GFP but with further extension of the π-π conjugation system to form an acylimine structure (Figure 1B). Therefore, as pH decreases, red FPs tend to decrease fluorescence by the same mechanism as GFP and YFP. Compared with GFP and YFP based proteins however, many more monomeric acid-tolerant RFPs have been reported (to the best of our knowledge, as to why RFP tends to have higher acid-tolerance than GFP has not been well studied). Among the reported RFPs, DsRed-derivatives such as mRFP1 (p*K*_a_ ≤ 4.5) [13], mCherry (<4.5) [14], mCherry2 (3.3) [15], mStrawberry (<4.5) [14], mScarlet (5.3) [16], mPlum (<4.5) [17] and mRaspberry (<4.5) [17], eqFP611-derivatives such as mRuby (4.4) [18], mRuby2 (5.3) [19] and mRuby3 (4.8) [20], and eqFP578-derivatives such as tagRFP (3.1) [21], TagRFP657 (3.4) [22], FusionRed (4.6) [23] are widely-used and highly pH-stable. DsRed- and eqFP611-derived FPs are likely resistant to proteolytic degradation in lysosomes, whereas eqFP578-derived FPs are not (the detail will be discussed in the following section). Therefore, in order to observe lysosome-related events, careful choice of FPs is necessary. For the application to acidic-organelles aside from lysosomes, FusionRed may be well-suited because of its superior monomeric property and less toxicity in comparison with other RFPs including mCherry and mRuby [23].

Large-Stokes-shift FPs (LSS-FP) such as mT-Sapphire (green) [24] and LSSmKate (red) [25] adopt a neutral Tyr side chain in their chromophore at steady state. When the neutral chromophore is excited, it converts to the excited intermediate anion form via proton transfer to a neighboring side chain of Asp/Glu (an example of so-called excited-state proton transfer (ESPT)) (Figure 1C). Red-shifted fluorescence is then emitted when excited electron (S^1^) transit to the ground state (S^0^). In the case of LSSmKate, pH sensitivity depends on the ionic state of Asp/Glu160 which act as a proton acceptor for the chromophore hydroxyphenol moiety via ESPT [26]. At pH levels below 3.5, the carboxylate group of Glu/Asp160 is protonated so that ESPT does not occur. Probably because p*K*_a_ of Glu/Asp side chains is low, LSSmKate1 and LSSmKate2 exhibit high acid-tolerance with p*K*_a_ of 3.2 and 2.7, respectively. 

### 2.2. Blue-Cyan Fluorescent Protein

Color variants that emit shorter wavelengths of light such as ECFP (cyan), EBFP (blue) and Sirius (ultramarine) were artificially constructed from *A. victoria*-derived GFP by substitution of the Tyr residue constituting the chromophore with a Trp, His or Phe residue, respectively [7]. The side chains of Trp, His and Phe in these chromophores are neutral or cationic state, in the β-barrel, at steady state, and environmental pH decrease causes a slight shrinkage of the π-π conjugation system as in the case of the GFP chromophore. Their fluorescence loss by pH decrease is mostly accompanied not with an absorption spectral shift but with a decrease in the fluorescence quantum yield. In this color range, several acid-tolerant FPs have been reported as follows. Sirius, a FP with Phe-derived chromophore emitting the shortest wavelength of excitation/emission peaks (355/424 nm), shows the highest acid-tolerance of all FPs, where its fluorescence intensity is essentially constant between pH 9.0 and 3.0 (p*K*_a_ < 3.0, Table 1) [27]. However, the molecular brightness is low (ε: 15,000 M^−1^·cm^−1^; QY: 0.24), and UV illumination to excite Sirius is likely to cause cytotoxicity. The best options so far are mTurquoise2 [30] and mCerulean3 [31], improved versions of ECFP with pH-insensitive indole rings. Their low p*K*_a_ values (3.1 and 3.2, respectively) and high molecular brightness (ε: 30,000 M^−1^·cm^−1^; QY: 0.93 for mTurquoise2; ε: 40,000 M^−1^·cm^−1^, QY: 0.80 for mCerulean3) make them amenable for use in acidic environments. Because of their high quantum yields, these FPs can serve as effective FRET donors for GFP and YFP. pH-tolerant Cyan-Green FP, ECGFP was also engineered from ECFP by substituting Thr underneath the chromophore with Tyr (T203Y) [33]. This substitution introduced π-π interaction between the chromophore’s indole ring and the Tyr’s phenolate ring, resulting in the shift of excitation/emission peaks from 434/476 nm to 463/506 nm. Another type of Blue FP, mTagBFP (and mTagBFP2) was developed by introducing mutations, which (1) prevent chromophore maturation from blue to red and (2) stabilize the blue protonated intermediate [28]. mTagBFP contains *N*-acylimine, the same chromophore as in TagRFP, but lacks a C^α^-C^β^ double bond in the Tyr side chain. mTagBFP2 shows the highest molecular brightness (ε: 50,600 M^−1^·cm^−1^; QY: 0.64) and acid tolerance (p*K*_a_ = 2.7) of all blue FPs.

### 2.3. Character and Stability of β-Barrel Structure

Chromophores from GFP-like proteins appear at first glance almost completely shielded by the rigid β-barrel structure. However, most GFP-like proteins have an unstructured loop region in the middle of the 7th β-strand, which makes a cleft-like structure between the 7th and 10th β-strand [42]. Some FPs such as Dronpa and TurboGFP have a water-filled opening there, and this connects the chromophore with bulk solvent. This opening may well be important for facilitating oxygen conveyance from the bulk solution to the premature chromophore, which may speed up the process of maturation [43]. Meanwhile, the opening possibly relays environmental pH changes to the chromophore, enhancing its pH sensitivity. Although the apparent opening has rarely been observed in the structure of other FPs, this opening at the same position may occur for other GFPs too. A molecular dynamics study has identified that fluctuation of the 6th–7th strand can generate an opening with enough size for water molecules passing through [44]. Also, X-ray crystallographic study of KillerRed revealed a water-filled channel at a different position reaching from an end cap of the β-barrel to the chromophore [45]. Therefore these are likely the reason why chromophores protected by rigid β-barrel structures are sensitive to pH. Resistance of the β-barrel structure itself to acidic quenching is also an important point. For example, at pH below 5.0, fluorescence intensity of EGFP and DsRed gradually decreases, and does not fully recover to the initial intensity after readjusting the pH to neutral. CD spectral measurement of DsRed revealed that the secondary structure breaks down in pH 4.1 buffer [46].

### 2.4. Resistance to Degradative Enzymes in Lysosomes

It has been observed that several days after transfection, some types of non-fused GFP-like proteins exhibit fluorescent puncta in cells, the localization pattern of which resembles that of lysosomes [29]. FPs in the cytosol are likely to be delivered to lysosomes by autophagy-related activity, then those resistant to both acidic quenching and degradation enzymes keep emitting fluorescence there. Incubation of several jellyfish-derived FPs (EGFP, ECFP and Sapphire) in pH 5.0 buffer containing lysosomal contents resulted in their loss of detection (relevant bands) by SDS-PAGE, while the same experiment with coral-derived FPs (such as DsRed, mRFP1 and zFP506) showed detection by SDS-PAGE at the expected size-positions. The control experiment using pH 5.0 buffer without lysosomal contents for both of the jellyfish- and coral-derived FPs also resulted in the appearance of the bands [29], together suggesting that only coral-derived FPs are resistant to acidic lysosomal degradation. For observation of events in the cytosol with FPs, this property is sometimes a problem because the fluorescent puncta may perturb analysis of imaging data and have a negative effect on long-term cell viability [39]. Meanwhile, FP resistance to degradation in lysosomes is essential for their application to lysosome-related imaging. For this purpose, mCherry and Gamillus are preferable because of their high resistance in lysosomes and their monomeric properties.

In addition, it has to be kept in mind that possible peptide cleavage may occur between the FP and the fused protein. It was reported that mCherry fused with the C-terminus of lysosomal proteins such as Niemann-Pick disease type C protein 2 (NPC2) and lysosomal protease tripeptidyl peptidase I (TPP1) showed separation of the two proteins due to lysosomal degradation enzymes [47]. This cleavage was suppressed by deletion of a flexible 11 amino acid region from the N-terminus of mCherry and the use of a rigid linker composed of 5 or 10 prolines. Therefore, to study trafficking of protein-complexes in lysosomes, which then travel from lysosomes to other organelles or plasma membrane, such linker optimization to prevent FP-fusion protein cleavage has to be considered.

## 3. FP-Based Sensor for Acidic Organelle Imaging

### 3.1. FP-Based Sensor and Chemical Dye-Based Sensor

To investigate the role of ions inside acidic organelles (e.g., Ca^2+^, Zn^2+^ and Cl^−^) and acidic organelle-related cellular events (e.g., autophagy, exocytosis and endocytosis), several organic dye-based and FP-based sensors have been developed (Table 2). Comparing the two types of sensor, organic dye-based sensors have advantages that include higher brightness, less photobleaching, easy sample preparation and superior resistance to acidic environments and degradative enzymes. The last characteristic is especially important for imaging applications in lysosomes. Accordingly, many organic dye-based sensors have been developed to obtain information about lysosomal ion concentration. Some organic dyes however are cytotoxic, (e.g., widely-used Ca^2+^ sensors such as Fluo-4 acetoxymethyl (AM), Rhod-2 AM and Fura-2-AM can suppress activity of Na^+^- and K^+^-dependent adenosine triphosphatase (Na,K-ATPase) [48]) so careful interpretation of data obtained with these dyes is necessary. In contrast, FP-based sensors have the advantage that they can be expressed in specific cell-types using a cell-selective promoter and can be easily localized to cellular compartments of interest by fusing them with organelle-specific targeting motifs or proteins. FP-based sensors, therefore, have been preferred for in vivo imaging [49], and imaging in specific organelles such as secretory granules, endosomes and Golgi body which are difficult for chemical dye-based sensors to correctly target (see Table 2). Their application in lysosomes is currently very limited due to the generally high pH sensitivity of FP fluorescence and their weak resistance to acidic quenching and proteolytic degradation.

**Figure 2:**
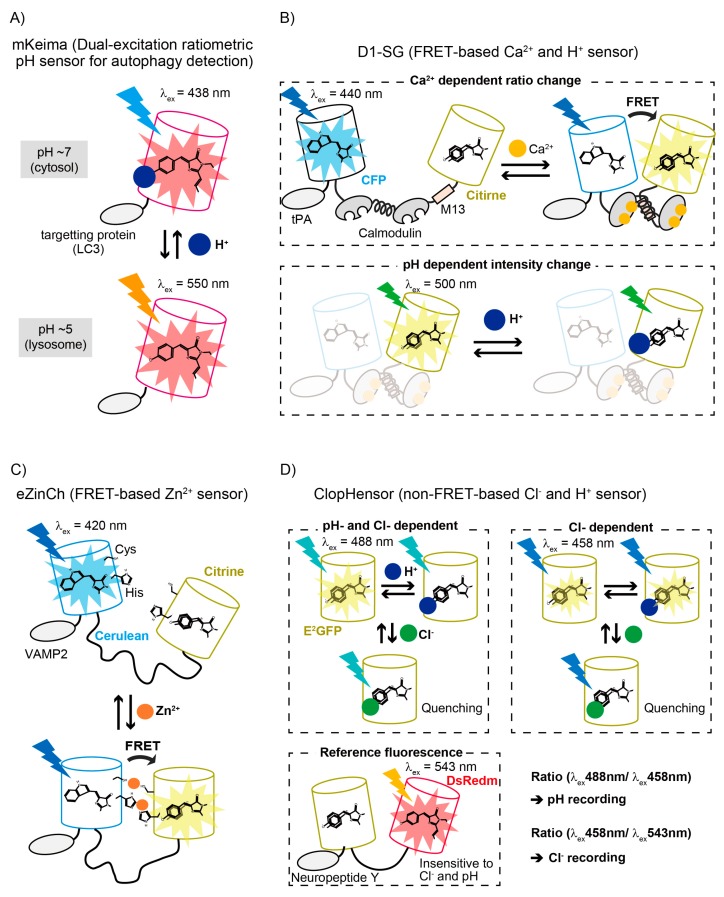
Schematic drawing of FP-based sensors for application in acidic organelles. (**A**) mKeima emits single emission at 620 nm, with dual excitation peaks at 438 and 550 nm, corresponding to protonated and deprotonated state of the chromophore. Under starvation-induced autophagy LC3-tagged mKeima is incorporated into autophagosomes, then recruited into lysosomes. This acidification induces color change of mKeima. LC3: microtubule-associated protein light chain 3; (**B**) D1-SG is a FRET-based and low-affinity Ca^2+^ sensor applicable at pH levels between 5.5 and 7.4 (*K*_d_ ~ 120–190 μM). Citrine can serve as an intentiometric pH sensor with direct excitation of ~500 nm, due to its inherent pH sensitivity (p*K*_a_ = 5.8). tPA: tissue plasminogen activator (secretory granule localization); (**C**) eZinCh is FRET-based, with low-affinity Zn^2+^ sensors composed of Cerulean and Citrine with Cys and His substitutions directed outside the β-barrel. Simultaneous use with pH sensor is necessary for pH calibration of the sensor. VAMP2: vesicle-associated membrane protein 2 (secretory granule localization); (**D**) ClopHensor records both Cl^−^ and pH in a ratiometric manner by applying three lasers for excitation (458, 488 and 543 nm). E^2^GFP changes the ratio of two excitation peaks and fluorescence intensity (protonated and deprotonated forms) depending on Cl^−^ concentration and pH. A pH isosbestic point exists at ~458 nm so that E^2^GFP emission under 458 nm excitation is pH-insensitive.

In the following section, we introduce several examples of the development and application of FP-based functional sensors in acidic organelles (Figure 2 and Table 2). Before moving to that topic, we explain how pH-dependent properties of FP-based sensors can introduce difficulties when investigating acidic organelles. We emphasize the importance of simultaneous measurement of pH values during imaging for calibration of the sensor. To the best of our knowledge, all ionic sensors are somewhat sensitive to pH changes (organic dyes are no exception). Additionally, placing the inherent pH sensitivity of FPs aside for a moment, the ionic state of functional binding domains in FP-based sensors, which are positively or negatively charged to trap their specific counter ions (e.g., Ca^2+^, Zn^2+^, Cl^−^), can be affected by pH changes. Generally, pH decreases results in decreased affinity for cation sensors and increased affinity for anion sensors. In addition, environmental pH alters electric charge on the protein surface (London forces [50] as well as ionic forces), which can change the ionic interaction between two FPs (in the case of FRET-based sensors) or that between the FP and the functional domain. pH values in acidic organelles are very dynamic in many cases, as listed here: (1) the maturation process from early endosomes to late endosomes (a pH drop from 6.3 to 5.5 [51]; (2) the starvation induced-pH drop in lysosomes which results in increased activity of lysosomal degradation enzymes to facilitate turnover of biomolecules [52]; (3) the fusion of several membrane vesicles (e.g., during formation of autolysosome); and (4) the release or collection of ions though channels or pumps on the membranes of acidic organelles accompanied with pH changes in order to balance ion gradients or for use in proton-motive force [53,54]. Therefore, discreet investigation of the pH-dependent property of sensors and careful pH-calibration of imaging data is essential to validate imaging results.

### 3.2. pH Sensors

A variety of FP-based pH sensors have been developed until now, including (1) intensity-based sensors composed of a single FP; (2) ratiometric-based sensors composed of a single FP and (3) FRET-based sensors composed of two FPs. Single FP-based ratiometric pH sensors allow more reliable measurement which is less affected by concentration changes of the sensors, and easy tagging into intracellular organelles because of their smaller size (~28 kDa) compared with FRET-based sensors. Thus, in this section, we mainly introduce some examples of single FP-based ratiometric pH sensors for pH measurement in acidic organelles. For details of other types of pH sensors, see other review articles, such as [55] published in 2013. In addition to various pH sensors listed in the article, new intentiometric RFP-based pH sensors, pHuji (p*K*_a_ = 7.7, Hill coefficient (n_H_) = 1.10) and pHoran4 (p*K*_a_ = 7.5, Hill coefficient (n_H_) = 0.92) have recently been reported [56]. 

mKeima is a large Stokes-shift version of monomeric red FP (Figure 2A) [57,58]. It exhibits a single emission peak (620 nm) with bimodal excitation spectra (438 and 550 nm peaks), corresponding to neutral (protonated) and anionic (deprotonated) forms of the chromophore, respectively. The ratio of the chromophore’s ionized form is dependent on pH with a p*K*_a_ of 6.5, thus mKeima can be used as a ratiometric pH sensor. mKeima also shows high resistance to lysosomal degradation enzymes. Katayama et al. utilized these advantages to develop a sensor to detect autophagic events, specifically conversion of autophagosomes to autolysosomes [58]. To achieve this, they fused mKeima with microtubule-associated protein light chain 3 (LC3). During starvation-induced autophagy, the LC3 undergoes cleavage and phosphatidylethanolamine modification, then being recruited to the outer and inner membrane of autophagosomes. Then, they fuse with lysosomes (autolysosomal maturation), so that the packed cargoes are exposed to an acidic and degradative environment for degradation. The mKeima-LC3 probe enabled visualization of these autolysosomal maturation events by detection of the acidification-induced color change of mKeima, and provided a cumulative fluorescent readout of autophagic activity, which was not possible with previous FPs undergoing acidic or proteolytic quenching in lysosomes. An improved version of the ratiometric dual-excitation pH sensor, pHRed was later developed from mKeima [59]. The ratio of fluorescence emission under excitation at 440 and 585 nm changes >10-fold dynamic range with an apparent p*K*_a_ of 6.6. pHRed also exhibits pH-dependent fluorescence lifetime in the range of pH 5 and 8 with near-infrared two-photon excitation. Because near-infrared light has higher light penetration and less light scattering in thick samples, such as brains, two-photon fluorescence lifetime imaging would offer an advantage for deep-tissue pH monitoring across a broad pH range.

E^1^GFP is a ratiometric dual emission pH sensor with a p*K*_a_ of 6.0 [60]. Under excitation at 403 nm, the emission peak shifts from 500 nm (pH ~ 5.0) to 523 nm (pH ~ 7.0). Although the mechanism is unclear, the authors expect it derives from a different acceptor residue in the ESPT process: presumably the neighboring E222 residue is protonated in low pH and cannot act as an ESPT acceptor. The utility to measure pH values in mildly-acidic organelles was demonstrated by fusing it with Trans-activating (Tat) viral protein and monitoring the process of intracellular trafficking after internalization in HeLa cells, which involved pH changes from ~6.3 to ~5.8.

### 3.3. Calcium Ion Sensors

D1-SG is a FRET-based sensor for simultaneous Ca^2+^ and pH measurement in secretory granules (Figure 2B) [65]. It was developed from a cameleon [86], which consists of a CFP and a Citrine (YFP) in conjunction with a calmodulin (CaM) insert that contains mutations to lower its Ca^2+^ affinity along with a CaM-binding peptide (M13) from myosin light-chain kinase. D1-SG also contains a tissue plasminogen activator (tPA) at the N-terminal for localization into secretory granules. In the presence of Ca^2+^ a CaM changes its conformation to interact with the M13 peptide (the CaM wraps around the M13 peptide). This conformational change renders the distance between the donor CFP and the acceptor Citrine closer, leading to an increase in the FRET efficiency. Surprisingly, Ca^2+^ affinity is hardly affected by pH changes between pH 7.4 and 5.5 (*K*_d_ ~ 120–190 μM), however, the FRET ratio value (CFP intensity to Citrine) is variable depending on pH. Therefore, simultaneous recording of pH values during the FRET measurement are required to calibrate the correct Ca^2+^ concentration. For use in secretory granules of PC12 cells, Citrine within D1-SG was directly excited to obtain an emission signal whose intensity was dependent on pH (p*K*_a_ = 5.8)—calibration was achieved by fully alkalizing the secretory granules by treating cells with NH_4_Cl solution (20 mM), whereby maximum emission was achieved. Accordingly, steady state pH and Ca^2+^ concentration in the secretory granules of PC12 cells was calculated as 5.8 and 69 ± 15 μM respectively. In the paper, Ca^2+^ imaging by using the D1-SG probe suggested important new roles for secretory granules, such as contributors to local cytoplasmic Ca^2+^ rise and refilling of luminal Ca^2+^ in the ER.

Single FP-based sensors such as the GCaMP series have been widely used to investigate Ca^2+^-related signal dynamics in the cytoplasm, especially in the field of neuroscience. GCaMP variants with low affinity to Ca^2+^, such as CEPIAs (*K*_d_ ~ 560–670 μM at pH 7.2) [87] and GCaMPer (*K*_d_ ~ 400 μM at pH 7.2) [88] have been developed to observe Ca^2+^ dynamics inside the endoplasmic reticulum. However, the application of most of the GCaMP series to acidic organelles is not currently practical due to their decreased fluorescence intensity at pH less than 7.0 [89]. Their application to imaging within lysosomes is almost impossible because most of them are not resistant to degradative enzymes. The fluorescence of DsRed and eqFP611-derived RCaMPs show higher resistance to degradation enzymes, although the resistance of the Ca^2+^-binding domain is not clear [39]. Among them, one good choice as a relatively pH-insensitive GCaMP-type sensor is GEM-GECO, which has been utilized to investigate the concentration and dynamics of Ca^2+^ in single endosomes [63,89]. GEM-GECO is composed of a circular permutant of GFP sandwiched with CaM and the M13 peptide. When excited at ~400 nm, GEM-GECO1 exhibits green (~510 nm) emission in the absence of Ca^2+^, and blue (~450 nm) emission in the presence of Ca^2+^. The dynamic range of GEM-GECO1 seems mostly unaffected by pH in the range of pH 7.50 and 5.42, although the Ca^2+^ affinity is reduced as pH drops (*K*_d_^Ca^ = 0.27 (pH 7.50), 0.46 (pH 7.00), 0.68 (pH 6.50), 3.09 (pH 6.00), 17.88 (pH 5.42), 54.24 μM (pH 4.97)). In order to correct for pH-dependent effects of the GEM-GECO’s Ca^2+^ affinity in endosomes, the authors used a ratiometric dual-excitation pH sensor, mKeima (p*K*_a_ = 6.0). Their semi-quantitative estimation by using the in vitro *K*_d_ values suggested that Ca^2+^ concentration within a majority of endosomes in MIN6 beta-cells is below 2 μM. Ca^2+^ concentration in more acidic Rab7-positive late endosomes was higher than that in Rab5a-positive early endosomes. Taken together, these Ca^2+^ sensors in combination with proper pH sensors will help researchers elucidate unknown mechanisms and roles of acidic organelles relating to the intracellular Ca^2+^ signaling network.

### 3.4. Zinc Ion Sensors

eCALWY and eZinCh are FRET-based sensors used to monitor intracellular free Zn^2+^ concentration (Figure 2C) [82]. For eCALWY, a functional Zn^2+^ binding domain composed of Atox1 and linked via a long flexible linker to domain 4 of ATP7B is sandwiched by Cerulean (CFP) and Citrine (YFP), both of which show reduced pH sensitivity. Mutations on both FPs at their dimeric interface (S208F and V224L) promoted an intramolecular interaction which increased FRET from CFP to YFP in the absence of Zn^2+^, while the interaction (and therefore the FRET) was decreased upon Zn^2+^ binding to the functional domain, leading to a 2.4-fold decrease in the emission ratio value. *K*_d_ (pH 7.1) for eCALWY variants 1–6 is 2, 9, 45, 630, 1850 and 2900 pM respectively, and *K*_d_ (pH 6.0) for VAMP2-eCALWY-6 is 0.5 μM. For eZinCh, Cerulean and Citrine with mutations (Y39H and S208C) to coordinate Zn^2+^ at their dimeric interface are connected via a long flexible linker. eZinCh exhibits *K*_d_ of 8 μM (pH 7.1) and 250 μM (pH 6.0). In the presence of Zn^2+^, Cerulean and Citrine presumably align in a parallel orientation to bind to Zn^2+^ at their respective Cys208, and subsequently bind to the 2nd Zn^2+^ at their respective His39, resulting in enhanced FRET efficiency [90]. These sensors could be targeted in insulin granules of INS-1 (832/13) cells by fusing them to the C-terminal of vesicle-associated membrane protein2 (VAMP2). As a result, free Zn^2+^ concentration in these vesicles was estimated at between 1 and 100 μM.

### 3.5. Chloride Ion Sensors

ClopHensor is a non-FRET-based sensor for simultaneous measurement of intracellular Cl^−^ concentration and pH (Figure 2D) [68]. It is composed of Cl^−^- and pH-sensitive E^2^GFP and Cl^−^- and pH-insensitive monomeric DsRed (RFP) linked via a long linker. E^2^GFP shows dual excitation spectra (~440 and 510 nm peaks) originating from neutral and ionic states of the chromophore, where the ratio is dependent on pH values with an isosbestic point at 458 nm. E^2^GFP also has a Cl^−^ binding pocket in the vicinity of the chromophore imidazolidinone aromatic ring and shows Cl^−^-dependent changes of the fluorescence properties. Cl^−^-binding to the protein does not affect the ratio of the neutral and ionic chromophore. Accordingly, (1) 458 nm excitation at the isosbestic point for E^2^GFP; (2) 488 nm excitation for E^2^GFP; and (3) 543 nm excitation for monomeric DsRed generate (1) Cl^−^-dependent; (2) pH- and Cl^−^-dependent; and (3) pH- and Cl^−^-independent signals. The pH calibration curve was obtained by calculating the ratio R_pH_ ((2) green to (1) cyan; p*K*_a_ = 6.81 ± 0.05 in Cl^−^ unligated form). The pH-dependent calibration curve for Cl^−^ concentration was derived from the ratio R_Cl_ ((1) cyan to (3) red; ^1^*K*_d_^Cl^ = 13.1 ± 0.5 mM in pH 6.9). The authors applied ClopHensor in large dense-core vesicles (LDCVs), the dominant secretory organelles in neuroendocrine cells by fusing to the N-terminal signal sequence of neuropeptide Y (NPY). They successfully revealed that the average pH of LCDVs is 5.2 ± 0.4 and the average Cl^−^ concentration is 110 ± 48 mM (mean ± s.d.).

## 4. Conclusions and Future Prospective

A variety of acid-tolerant FPs (p*K*_a_ < 4.0) with a wide color-range of excitation and emission spectra have been developed. These provide researchers with more options to perform multicolor imaging and develop single FP-based and FRET-based sensors to elucidate molecular or ionic mechanisms in acidic cellular environments [91]. Many different ions inside acidic organelles play important roles during regulation of the activity of internal proteins, cellular signaling and biological homeostasis. To investigate their functions, several ion sensors (Ca^2+^, Zn^2+^ and Cl^−^) have been developed and applied in acidic organelles. As we discussed in Section 3, simultaneous use of a pH sensor for pH calibration is essential for quantitative measurement. In order to avoid spectral overlap, good combinations of ion and pH sensor are as follows: (1) a FRET-based ion sensor composed of CFP and GFP (or YFP), and an intentiometric or ratiometric pH sensor composed of a single RFP (e.g., mOrange2, mKeima and pHRed [40,57,59]); (2) a ratiometric ion sensor composed of a single GFP (or YFP), and an intentiometric or ratiometric pH sensor composed of a single RFP; (3) a ratiometric ion sensor composed of a single RFP, and an intentiometric or ratiometric pH sensor composed of a single GFP (e.g., mNeonGreen and E^1^GFP [37,60]); (4) a FRET-based ion sensor in which acceptor fluorescence is pH sensitive upon direct excitation (e.g., D1-SG, Figure 2B [65]); (5) an exceptional FP which shows bimodal excitation (or emission) spectra sensitive to both specific ion and pH (e.g., ClopHensor, Figure 2D [68]), and a FP insensitive to both the ion and pH which is used as reference fluorescence for ratiometric measurement. 

Among the various ion species in acidic organelles, in particular Ca^2+^-mediated communication between acidic organelles and neutral pH organelles, as well as signaling network activation triggered by Ca^2+^ release from acidic organelles in both mammalian and plant cells have recently become areas of increased interest for researchers [4]. The vacuole is the largest and acidic Ca^2+^-store in plant cells, which may contribute to broad Ca^2+^ signaling, however, precious few functional roles have been directly demonstrated [92]. The development of Ca^2+^ sensors with less pH sensitivity would be highly useful for investigating these temporal and spatial dynamics. New types of FP-based sensors to monitor cytosolic K^+^ (GEPIIs and KIRIN-1 [93,94]) and Mg^2+^ concentration (MagFRET and MARIO [95,96]) have recently been published. Improvement of these sensors to facilitate their application to acidic cellular environments may help to reveal heretofore unknown functions (e.g., ion homeostasis, ionic gradients or potential across the membrane, and subsequent biological events). Sensors to detect amino acids such as glutamate (SuperGluSnFR, iGluSnFR, iGlu_f_ and iGlu_u_ [97,98,99]) and arginine (QBP/Citrine/ECFP [100]) have also been reported. The development of such sensors for acidic environments would be useful, especially in the case of glutamate release from synaptic vesicles (pH ~ 5.8 [64]) into the synaptic cleft, which induces neural excitation; the concentration and turnover rate of glutamate into the synaptic vesicles would help in the study of synaptogenesis. The development of a lysosomal arginine sensor would be useful to unveil potential connections between arginine concentration in lysosomes and the autophagy-related signaling pathway [101]. As an alternative application for imaging with acid-tolerant FPs, the development of new acid-tolerant chemiluminescent proteins (CPs) will also expand choices for researchers: CPs can overcome flaws inherent to FPs due to their requirement for excitation light including autofluorescence from samples, undesired activation of light-dependent biological process such as photoreception, and incompatibility with optogenetic tools due to their spectral overlap [102]. To date, there are no acid-tolerant CPs optimized for imaging application. Protein engineering of unique dinoflagellate-derived CPs which emit strong luminescence in acidic conditions [103], or searching for new CPs from uncharacterized bioluminescent species will fulfill these niche but necessary demands of researchers in the future.

## Figures and Tables

**Table 1:** Optical property of acid-tolerant GFP-like proteins.

Color	Protein	λ_ex/em_^a^ (nm)	ε ^b^ (10^3^ M^−1^ cm^−1^)	QY ^c^	Bright-ness ^d^	Oligomeric State ^e^ (In Vitro/OSER)	p*K*_a_^f^	Resistance to Lysosomal Enzymes	Ref.
Blue	Sirius	355/424	15.0	0.24	3.6	mono/N.D.	<3.0	Yes ^g^	[27]
	mTagBFP2	399/454	50.6 (76.0)	0.64 (0.48)	32.4 (36.5)	mono/oligo	2.7 (2.4)	eqFP578-derivatives. Maybe no.	[28]
Cyan	mTurquoise2	434/474	30.0 (31.0)	0.93 (0.92)	27.9 (28.5)	mono/mono	3.1 (3.6)	avGFP-derivatives. Maybe no. [29] ^h^	[30]
	mCerulean3	434/475	40.0 (29.0)	0.80 (0.80)	32.0 (23.2)	mono/mono	3.2 (3.4)		[31]
	mTFP1	462/492	64.0 (53.0)	0.85 (0.85)	54.4 (45.1)	mono/mono	4.3 (4.3)	N.D.	[32]
UV-excitable Green	mT-Sapphire	399/511	44.0 (34.0)	0.60 (0.59)	26.4 (20.1)	mono/mono	4.9 (4.8)	avGFP-derivatives. Basically no.	[24]
Green	ECGFP	463/506	23.9	0.14	3.3	mono/N.D.	<4.0		[33]
	mEmerald	487/509	57.5 (62.0)	0.68 (0.75)	39.1 (46.5)	mono/mono	6.0 (4.6)		[34]
	pH-tdGFP	488/515	N.D.	N.D.	N.D.	tandem-dimer	4.8		[35]
Yellow-Green	mVenus	515/527	105.0 (127.0)	0.64 (0.67)	67.2 (85.1)	mono/mono	6.0 (5.4)		[36]
	Gamillus	504/519	83.0	0.90	74.7	mono/mono	3.4	Yes	[12]
	mNeonGreen	506/517	116.0 (113.0)	0.80 (0.80)	92.8 (90.4)	mono/mono	5.7 (5.4)	Maybe no [12]	[37]
Orange	mKOk	551/563	105.0	0.61	64.1	mono/N.D.	4.2	N.D.	[38]
Red	mRFP1	584/607	50.0 (55.0)	0.25 (0.35)	12.5 (19.3)	mono/mono	4.5 (3.8)	DsRed-derivatives. Basically yes. [39]	[13]
	mCherry	587/610	72.0 (85.0)	0.22 (0.30)	15.8 (25.5)	mono/mono	<4.5 (3.8)		[14]
	mCherry2	589/610	79.4	0.22	17.5	mono/N.D.	3.3		[15]
	mStrawberry	574/596	90.0	0.29	26.1	mono/mono	<4.5		[14]
	mScarlet	569/594	100.0	0.70	70.0	mono/mono	5.3		[16]
	mRuby	558/605	112.0 (109.0)	0.35 (0.38)	39.2 (41.4)	mono/mono	4.4 (4.4)	eqFP611-derivatives. Basically yes. [39]	[18]
	mRuby2	559/600	113.0 (107.0)	0.38 (0.37)	42.9 (39.6)	mono/mono	5.3 (4.4)		[19]
	mRuby3	558/592	128.0	0.45	57.6	mono/N.D.	4.8		[20]
	TagRFP	555/584	100.0 (130.0)	0.48 (0.33)	48.0 (42.9)	mono/oligo	3.1 (3.0)	eqFP578-derivatives. Basically no. [39]	[21]
	TagRFP-T	555/584	81.0 (106.0)	0.41 (0.32)	33.2 (33.9)	mono/oligo	4.6 (4.3)		[40]
	FusionRed	580/608	83.0 (85.0)	0.19 (0.30)	15.8 (25.5)	mono/mono	4.6 (4.2)		[23]
Blue-excitable Red	LSSmKate1	463/624	31.2	0.08	2.5	mono/N.D.	3.2		[25]
	LSSmKate2	460/605	26.0	0.17	4.4	mono/N.D.	2.7		[25]
	hmKeima4.15	436/612	28.0	0.29	8.1	mono/N.D.	5.2	mKeima-derivative. Maybe yes.	[41]
Far Red	mPlum	590/649	41.0 (80.0)	0.10 (0.13)	4.1 (10.4)	mono/mono	<4.5 (4.6)	DsRed-derivatives. Maybe yes.	[17]
	mRaspberry	598/625	86.0	0.15	12.9	mono/mono	<4.5		[17]
	TagRFP657	611/657	34.0	0.10	3.4	mono/N.D.	3.4	eqFP578-derivatives. Maybe no.	[22]

^a^ Wavelength of excitation and emission peaks; ^b^ Molar extinction coefficient; ^c^ Fluorescence quantum yield; ^d^ Product of ε and QY, divided by 1000; ^e^ Oligomeric state of FPs determined by gel-filtration chromatography (in vitro) or OSER assay with mammalian cells; ^f^ pH at which fluorescence intensity becomes its half-maximal value; ^g^ Sirius is derived from avGFP, however it kept emitting fluorescence in phagosomes in *Dictyostelium discoidium*; ^h^ FPs from the same origin tend to show similar resistance to proteolytic degradation in lysosomes. Values without parentheses are from original articles. Values indicated in parentheses are referred from a paper [8]. N.D. indicates “Not Determined”.

**Table 2:** FP-based or chemical dye-based sensors for measurement of ion concentration in acidic organelles.

Ion	Organelle	Cell	Sensor	Class	p*K*_a_ or *K*_d_ (pH or p*K*_a_)	Resting pH, or [Ca^2+^, Zn^2+^ or Cl^−^] (pH)	Ref.
pH	Endosome	Foreskin keratino-cyte	Cellubrevin-r-pHluorin	Gen ^a^, Single FP-base dual-excitation	6.9 [61]	Early endosome: 5.9	[62]
		HeLa	Tat-E^1^GFP	Gen, Single FP-base dual-excitation	6.4–6.7	Early endosome: 6.8 Endosome: 5.8–6.3	[60]
		MIN6 beta-cell	TiVAMP-mKeima	Gen, Single FP-base dual-excitation	5.8	Early endosome: 6.3 Late endosome: 5.8	[63]
	Synaptic vesicle (SV)	Hippocam-pal neuron	Synaptophysin-mOrange2	Gen, Single FP-base, intensiometric	6.5	GABAergic SV: ~6.4 Glutamatergic SV: ~5.8	[64]
	Secretory granule	PC12	Citrine (YFP) used in D1-SG (Ca^2+^ sensor)	Gen, intensiometric	5.8	5.8	[65]
		PC12	CgA-ECFP	Gen, Single FP-base, FLIM	N.D.	5.5	[66]
		MIN6	VAMP2-pH.fluorin(e)	Gen, Single FP-base, Intensiometric	N.D.	6.3	[67]
		PC12, WSS-1	NPY-ClopHensor	Gen, Ratiometric (Non-FRET-base)	6.8	5.2 (PC12) 5.6 (WSS1)	[68]
	Lysosome	HeLa, MCF-7	Lyso-pH	Chem ^b^, Ratiometric	5.0	4.6	[69]
	Vacuole	*A. thaliana*	Aleurain-PRpHluorin	Gen, Single FP-base dual-excitation	6.6	5.2	[70]
		*A. niger*	RaVC (improved version of pHluorin for pH imaging in filamentous fungi)	Gen, Single FP-base dual-excitation	6.7 (in vitro) 6.9 (in cell)	6.2–6.5	[71]
Ca^2+^	Endosome	MIN6 beta-cell	TiVAMP-GEM-GECO1	Gen, Single FP-base dual-emission	0.27 (pH 7.5), 0.46 (7.0), 0.68 (6.5), 3.1 (6.0), 17.9 (5.4), 54.2 μM (5.0)	Early endosome: 0.5 (6.3) Late endosome: 2.5 μM (5.8)	[63]
		3T3 Swiss fibroblast	Oregon green 488 BAPTA-5N	Chem, Intensiometric	20 μM	3.0 μM (5.7) (30 min incubation)	[72]
		Pancreatic acinar cell	Oregon green 488 BAPTA-5N	Chem, Intensiometric	36.5 (pH 7.2), 55.3 (5.9), 116 μM (5.3)	37 μM (5.9)	[73]
	Secretory granule	PC12	D1-SG	Gen, FRET-base	120–190 μM (pH 7.4–5.5)	69 μM (5.8)	[65]
		MIN6	VAMP2-mut.aequorin	Gen, Intensiometric	1–10 μM	~50 μM (6.3)	[67]
		PC12	Chromogranin-aequorin	Gen, Intensiometric	3.8 μM [74]	1.4 μM (~5.5)	[75]
		Sea urchin egg	Fluo-4	Chem, Intensiometric	345 nM (pH 7.2) [76]	~10–100 μM	[77]
	Lysosome	Mouse macrophage	Fura-2 dextran	Chem, Ratiometric	~200 μM	600 μM (4.5)	[53]
		Mouse macrophage	Oregon green BAPTA-1 dextran	Chem, Intensiometric	~500 μM	400 μM (4.5)	[53]
		Human fibroblast	Rhod dextran	Chem, Intensiometric	551 ± 107 μM	550 μM (4.5)	[78]
Cl^−^	Endosome	J774 cell, CHO cell	BAC-TMR-dextran	Chem, Ratiometric	~25–50 mM (pH 7.4)	17→53 mM (6.95→5.30) (J773) 28→73 mM (6.92→5.60) (CHO)	[79]
		J774 cell, CHO cell	BAC-dextran-Tf-TMR	Chem, Ratiometric	~25–50 mM (pH 7.4)	18→40 mM (6.91→6.05) (J773) 24→46 mM (6.95→6.18) (CHO)	[80]
	Secretory granule	PC12, WSS-1	NPY-ClopHensor	Gen, Ratiometric (Non-FRET-base)	13.1 mM (p*K*_a_ 6.81)	110 ± 48 mM (5.2) (PC12) 122 mM (5.6) (WSS1)	[68]
	Lysosome	Venticular myocyte	6-methoxyquino-linium–dansyl	Chem, Ratiometric	~15 mM (pH 4.5)	9.46 mM	[81]
Zn^2+^	Secretory granule	INS-1 (832/13)	VAMP2-eZinCh	Gen, Ratiometric (FRET)	8 (pH 7.1), 260 μM (6.0)	1–100 μM	[82]
	Lysosome	NIH 3T3	DQZn4	Chem, Ratiometric	16 nM (pH 5.2)	N.D.	[83]
		NSCs, MCF-7, HeLa	LysoZn-1	Dhem, Ratiometric	~150 μM (pH 7.2)	N.D.	[84]
		NIH 3T3	probe 1 (no name)	Chem, Intensiometric	8.55 μM (pH 5.0)	N.D.	[85]

^a^ FP-based sensor; ^b^ Organic dye-based sensor.
